# EFFECT OF NECK-SPECIFIC EXERCISES WITH AND WITHOUT INTERNET SUPPORT ON CERVICAL RANGE OF MOTION AND NECK MUSCLE ENDURANCE IN CHRONIC WHIPLASH-ASSOCIATED DISORDERS: ANALYSIS OF FUNCTIONAL OUTCOMES OF A RANDOMIZED CONTROLLED TRIAL

**DOI:** 10.2340/jrm.v56.34785

**Published:** 2024-07-29

**Authors:** Gunnel PETERSON, Emma NILSING STRID, Margaretha JÖNSSON, Jesper HÄVERMARK, Anneli PEOLSSON

**Affiliations:** 1 Centre for Clinical Research Sörmland, Uppsala University, Eskilstuna; 2 Department of Health, Medicine and Caring Sciences, Physiotherapy, Linköping University, Linköping; 3 University Health Care Research Center, Faculty of Medicine and Health, Örebro University, Örebro; 4 Centre for Clinical Research, Development and Education, County Council Uppsala; 5 Occupational and Environmental Medicine Centre and Department of Health, Medicine and Caring Sciences, Unit of Clinical Medicine, Linköping University, Linköping, Sweden

**Keywords:** telemedicine, rehabilitation, whiplash injuries, neck pain, spine

## Abstract

**Objective:**

To compare the effects of a neck-specific exercise programme with internet support and 4 physiotherapist sessions (NSEIT) and the same neck-specific exercises supervised by a physio-therapist (NSE) on neck muscle endurance and cervical range of motion.

**Design:**

Randomized controlled trial.

**Patients:**

A total of 140 participants with chronic whiplash-associated disorders grade II or grade III were randomly assigned to the NSEIT or NSE groups.

**Methods:**

Outcomes were changes in active cervical range of motion, cranio-cervical flexion test, neck muscle endurance, and neck pain, at 3- and 15-month follow-ups.

**Results:**

There were no significant differences between the NSEIT and NSE groups. There was a significant group-by-time interaction effect in active cervical range of motion flexion/extension where the NSEIT group improved to 3-month follow-up, but the NSE group did not. Both groups were significantly improved over time in all other outcomes (*p* < 0.001) at 3- and 15-month follow-ups, with effect size between 0.64 and 1.35 in active cervical range of motion, cranio-cervical flexion test, dorsal neck muscle endurance, and neck pain, and effect size between 0.22 and 0.42 in ventral neck muscle endurance.

**Conclusion:**

Both NSE and NSEIT led to improved neck function. Depending on the patients’ needs, either NSE or NSEIT could be used as treatment for patients with chronic whiplash-associated disorders.

## LAY ABSTRACT

Chronic disability and pain are common after whiplash injury, but effective rehabilitation methods are lacking. A neck-specific exercise (NSE) programme has shown promising results in terms of decreased pain and disability after 12 weeks of supervised neck-specific exercise at a physiotherapy clinic. Fewer face-to-face meetings with internet-based exercises may save time and costs for both patients and healthcare services, but this needs to be evaluated. In this study, we compared the effect of internet-delivered neck-specific exercise (NSEIT) with 4 physiotherapist sessions and supervised NSE on neck movement and neck muscle endurance. The results showed that NSEIT and NSE improved neck movement, muscle endurance, and neck pain after muscle endurance tests to the same extent. The results were sustained at 15-month follow-up, and NSE or NSEIT could be used to treat chronic whiplash, responding to the need for increased flexibility and availability of effective exercise interventions.

Persistent disability and neck pain are common after motor vehicle accidents, with up to 50% of those who are injured having symptoms years after the accident (1–3), so-called whiplash-associated disorders (WAD). Whiplash injury is the most frequent injury in motor vehicle accidents and affects 43.8% of individuals involved in such accidents (2). Improving neck muscle endurance and function is essential after whiplash injury, as individuals with WAD have neck muscle weakness (4), more restricted cervical range of motion (5), low neck muscle endurance, and more disability (6) compared with patients with non-traumatic neck pain. Interventions including exercises and education are recommended (7), but there is still no clear evidence of benefit from exercise interventions for chronic WAD (8–12), highlighting the importance of finding effective exercise methods. In WAD grade I (neck pain and no physical symptoms) and grade II (neck pain and physical symptoms after the accident), advice was just as effective as an exercise programme (8, 12). Aerobic exercises were more effective to decrease pain intensity compared with strengthening exercises in a small single case experimental study (9). Combined exercise and neuroscience education was effective to reduce pain and disability in chronic spinal pain, including whiplash-injured patients (11), but with only small to moderate effect size. An NSE programme showed better results compared with a general exercise intervention (10) and control group (13) in WAD grade II and grade III (neck pain and physical and neurological symptoms). Decreased neck pain in WAD after NSE was related to improved neck muscle endurance (14), and NSE was especially important for those with low neck-related function, as a more general exercise regime caused increased disability and pain (15). Moreover, improved interaction was seen between deep and superficial ventral neck muscles after NSE (16). NSE included visits twice a week for 12 weeks, totalling 24 visits to a physiotherapist, and more effective ways of delivering NSE are needed. Internet-administered exercise intervention may save time and costs (17) for both patients and healthcare services, and blended intervention with both digital and face-to-face meetings was preferred for patients with dizziness (18). To our knowledge, no studies have investigated whether internet-based exercises can improve neck function in persistent neck pain.

The aim of the present study was to evaluate the effects on neck muscle endurance and cervical range of motion by comparing 2 different ways to deliver neck-specific exercises: an internet-supported neck-specific exercise programme (NSEIT) versus the same exercises (NSE) performed at a physiotherapy clinic.

## METHODS

Participants were included in a prospective randomized controlled (RCT) multi-centre study comparing NSEIT vs supervised NSE with 3- and 15-month follow-ups (19, 20). The study was approved by the regional ethical review board in Linköping, Sweden (2016/135-31) and followed the Declaration of Helsinki. The study protocol has been published elsewhere (19) and the trial was registered with ClinicalTrials.gov (NCT03022812) before data collection started (first posted date 18/01/2017). The participants were recruited from 10 counties in Sweden between 6 April 2017 and 15 September 2020, and written informed consent was obtained from all participants before allocation. The present study was a planned analysis of secondary outcomes of active range of motion (ACROM), cranio-cervical flexion test (CCFT), and neck muscle endurance (NME).

### Participants and randomization

The recruitment of participants and the exercise interventions were reported earlier (20), and are briefly described below. Information approaching the study was provided by healthcare providers, via advertisements in newspapers, social media, and the university’s website. Interested participants answered a short online survey including questions regarding inclusion criteria: neck symptoms associated with a whiplash injury at least 6 months but not more than 5 years prior to study entry, age between 18 and 63 years, average neck pain intensity over the past week > 20 mm according to a 100-mm visual analogue scale (VAS), and > 20 on the Neck Disability Index (NDI, 0–100). The next step involved a telephone interview to further verify their ability to participate in the exercise programme and a medical history review to confirm inclusion and exclusion criteria. To be included, the participants also should have: daily access to a computer, tablet, or smartphone and the internet; time to follow the treatment programme; and symptoms of neck pain, neck stiffness, or cervical radiculopathy within the first week after the injury. Exclusion criteria were: head injury at the time of the whiplash injury, including loss of consciousness, amnesia before or after the injury, altered mental status (e.g., confusion or disorientation), or focal neurological changes in smell and taste; previous fractures or dislocation of the cervical spine; known or suspected serious physical pathology; spinal tumours, spinal infection, or ongoing malignancy; previous severe neck problems that resulted in sick leave for more than a month in the year prior to the current whiplash injury; cervical spine surgery; generalized or more dominant pain elsewhere in the body; other illness/injury that may prevent full participation; inability to understand and write in Swedish; diagnosed severe mental illness, such as psychosis, schizophrenia, or personality disorders; current alcohol or drug abuse (20); or participation in the earlier NSE study (10). The last step in recruiting was a physical examination conducted by a test leader (physiotherapist) to further confirm study inclusion, i.e., WAD grade II or III (19, 20).

A computer-based block randomization list stratified by sex was used for randomization to the 2 groups, and the participant’s group allocation and contact details were sent to the treating physiotherapist in a sealed opaque envelope. The test leaders were blinded to group allocation at baseline and at 3- and 15-month follow-ups. The participants were blinded to group allocation when baseline data were collected.

### Interventions

Both interventions were reported earlier (20), and are briefly described below. Experienced physiotherapists delivered the exercise programme, and they received a day of standardized theoretical and practical training from the research team prior to study commencement.

The NSEIT group had 4 face-to-face visits to a physiotherapist. The first session included a clinical examination and an introduction to the first exercises. The follow-up sessions at weeks 2, 3, and 7 included follow-up of exercises, progression, and introduction of new exercises. Participants had access to the internet-based programme via a website, and the programme included photos and videos of all the exercises as well as information regarding the whiplash injury mechanism, the relevant musculoskeletal anatomy and function, neurophysiological and neurobiological processes underlying chronic pain, and the aim of the exercise programme to activate deep neck muscles and thereafter improve global neck muscle endurance, as well as an instruction that the patient should draw up a plan for neck pain relapse during the last session.

The NSE group received the same information and exercise programme as the NSEIT group, but delivered by a physio-therapist. The participants had a total of 24 face-to-face visits to the physiotherapist (2 sessions a week for 12 weeks). Exercise-related neurological pain was not acceptable in NSEIT or NSE, and increased muscle soreness related to neck-specific exercises was allowed only if it did not increase neck pain over time. The physiotherapist needs to adjust the exercises if patients have experienced neurological pain and/or increased neck pain over time. In the event of continued problems, further examinations should be undertaken to determine whether the exercises should be stopped.

Participants in both groups were encouraged to continue training after the 12 weeks on their own in accordance with the 2017 World Health Organization guidelines (21) and to include NSE in their training programme.

The exercise programme was the same as in our previous study (10), except that 2 ventral neck exercises were added after approximately 4–6 weeks of initial exercises. These 2 exercises were performed in a supine position and in the first exercise patients performed a slight nod without lifting the head. The second exercise was a progression: the participants performed a slight nod and lifted the head 2–3 cm. All the included exercises can be seen in Peterson and Peolsson (20). The reason for the addition was that more than 50% of participants in our previous RCT did not achieve the lower limits for normal reference values in ventral NME, which are ≥ 23 s for women and ≥ 56 s for men (15, 22).

### Outcomes

All measurements followed a strict test protocol and were taken in the same order: active cervical range of motion (flexion, extension, lateral flexion, and rotation), deep ventral neck muscle endurance, and global ventral and dorsal neck muscle endurance. The participants reported their neck pain intensity immediately before and after the tests. Baseline characteristics were reported using questionnaires on Linköping University’s website Survey and Reports.

Active cervical range of motion (ACROM) was assessed in sagittal (flexion/extension), frontal (lateral flexion), and transverse (rotation) planes using a cervical range of motion (CROM) device, showing substantial reliability (intraclass correlation coefficient [ICC]; 0.77 to 0.99) (23). The CROM device was placed on the participant’s head when sitting upright with neutral pelvic position, hips and knees at 90°, and arms resting in the lap. The participants were told to move their head as far as possible, keeping the body and shoulders still, and the test leader held their hands on the participants’ shoulders to minimize movements in the body and shoulders. At the end of each movement, the participants took a short pause so readings could be taken. They then returned to the neutral position before the next movement.

Deep ventral neck muscle activation was assessed using the cranio-cervical flexion test (CCFT). The CCFT has shown substantial intra- and inter-examiner reliability (ICC; 0.63 to 0.86) (24). The CCFT was measured after ACROM. A pressure biofeedback, Stabilizer (Chattanooga Group Inc., Austin, TX, USA), was placed so that it abuts the occiput with the participants in supine lying position, with their arms alongside their body and the cervical spine in a neutral position. The Stabilizer was inflated to 20 mmHg, and the participants performed a cranio-cervical flexion to 22 mmHg and held the position for 5 s without activation of superficial ventral neck muscles (sternocleidomastoid and/or m scalenus), before returning to 20 mmHg. The participants performed cranio-cervical flexion through 5 progressive stages (22–30 mmHg). The test leader detects the presence of any activity in the superficial flexors by visual inspection and a light touch of their hand. The test was stopped if activation of the superficial muscles occurred, and the level below was registered.

The global ventral and dorsal neck muscle endurance test (NME) has good reliability (ICC 0.88–0.93) (25), and was measured in seconds and standardized as previously described (26). The ventral NME was measured after the CCFT test. Ventral NME was tested with the patient in a supine position, with their arms alongside their body and cervical spine in a neutral position. The participants were instructed to nod slightly, retract their chin, and raise their head just above the examination table. The participants practised the test once before the test started, lying supine with a slight nod, and retracting the chin but not lifting the head.

Dorsal NME was measured after ventral NME, with the patient in a prone position, positioned with straight legs, arms alongside the body, and head initially supported on the examination table. A load (2 kg for females and 4 kg for males) was applied to the head, and the participants were instructed to lift their head just above the examination table. The tip of the chin should point at the floor, thus performing a slight extension of the cervical spine. Before the test started, dorsal NME was practised once with the chin pointing at the floor and head lifting without the weight.

The test position in both ventral and dorsal NME should be maintained for as long as possible. The test position was corrected by the test leader if necessary, and the test was stopped if the participant was unable to hold the test position. The participant was instructed to stop the test by returning their head to rest at the point of neck fatigue, or in the event of pain radiating into the arm or severe neck pain.

### Neck pain intensity

Pain intensity was measured using the visual analogue scale (VAS; 0 mm [no pain] to100 mm [worst imaginable pain]) immediately before and after the tests (27).

### Statistics

The statistical analyses were performed using IBM SPSS Statistics for Windows (Version 28.0. IBM Corp, Armonk, NY, USA). Between-group differences at baseline were analysed with independent samples *t*-test for continuous normally distributed variables and with Pearson’s χ^2^ or Fisher’s exact test for categorical variables (Table I). Sample size and power calculations were based on the primary outcome Neck Disability Index (NDI) (19, 20), and 47 participants were needed in each group to detect a difference of 7% in NDI. To account for attrition, 70 participants were included in each group.

**Table I:** Baseline descriptive characteristics of trial participants, by treatment group

	NSEIT^a^ (*n* = 70)	NSE^b^ (*n* = 70)
Age, mean (SD)	40.4 (11.6)	40.5 (11.4)
Months since injury, mean (SD)	27.4 (21.0)	25.2 (15.5)
WAD grade, *n* (%):		
Grade II	46 (66)	43 (61)
Grade III	24 (34)	27 (39)
Sex, *n* (%):		
Female	55 (79)	55 (79)
Male	15 (21)	15 (21)
Education, *n* (%):		
Elementary	1 (1)	0
High school	30 (43)	39 (57)
University	35 (50)	27 (39)
Other	4 (6)	3 (4)
Marital status, *n* (%):		
Married or cohabiting	52 (74)	53 (76)
Single	18 (26)	17 (24)
Previous treatment, yes, *n* (%)	61 (87)	64 (91)
Physiotherapist primary care	37 (52)	44 (62)
Physiotherapist specialist care	16 (22)	24 (34)
Physiotherapist occupational health care	17 (24)	22 (31)
Chiropractic	32 (46)	32 (46)
Pain rehabilitation clinic	16 (22)	23 (33)
Previous treatment, *n* (%):		
Advice	30 (43)	23 (33)
Neck exercises^b^	37 (53)	36 (51)
General exercises	15 (21)	12 (17)
Massage	0	4 (6)
Acupuncture	10 (14)	9 (13)
Other (e.g., osteopathy, yoga, manipulation)	5 (7)	8 (11)
Expectation from participating in the study, *n* (%):		
Fully recovered	3 (4)	5 (7)
Much improved	51 (73)	49 (70)
Some relief	14 (20)	15 (22)
No expectations	2 (3)	1 (1)
Use of analgesic drugs, yes, *n* (%)	57 (81)	59 (84)
Compensation, *n* (%)		
No	22 (31)	15 (21)
Yes	29 (42)	42 (60)
Not decided	19 (27)	13 (19)
Present employment status, *n* (%):		
Employed, full-time/part-time	38 (54)/14 (20)	44 (63)/12 (17)
Self-employed, full-time/part-time	3 (4)/6 (8)	4 (6)/2 (3)
Unemployment compensation, full-time	1 (1)	1 (1)
Unemployed, no compensation, part-time	1 (1)	1 (1)
Student, full-time	7 (10)	7 (10)
Sick-leave, full-time/part-time, *n* (%)	3 (4)/6 (9)	3 (4)/5 (7)

Data are number (%) of participants unless stated otherwise.

a Data are mean (standard deviation).

b Neck-specific exercises other than the exercises in the present study.

NSEIT: neck-specific exercises with internet support; NSE: neck-specific exercises supervised at a physiotherapy clinic. There were no significant differences in baseline variables between the groups, *p* ≥ 0.15.

The analyses were conducted on an intention-to-treat basis. Due to the presence of missing data in outcome measures at 3- and 15-month follow-ups, we conducted missing pattern analyses on each outcome measure, comparing completers and non-completers (participants with missing data at 3- and/or 15-month follow-up) both within and between the NSEIT and NSE groups, using an independent samples *t*-test. In addition, between-group comparison of the proportion of completers in each outcome measure was analysed with Pearson’s χ^2^ test.

Linear mixed models (LMMs) were used to analyse repeated measures of CCFT, NME, ACROM, and pain intensity, with time points (baseline, 3 months, 15 months) and intervention group (NSEIT, NSE) treated as fixed effects. Restricted maximum likelihood estimate was used in the LMMs, allowing all participants with at least 1 observation to be included in the models, under the assumption of data missing at random. The unstructured covariance structure was applied in all models to measure the association among the repeated measures. Sex (men, women) and age were entered as covariates in the models. Bonferroni correction was applied on all multiple pairwise contrasts between the three timepoints. Cohen’s *d* effect size was calculated for all significant fixed effects and interpreted as *d* > 0.2 = a small effect, *d* > 0.5 = a medium effect, and *d* > 0.8 = a large effect (28) (Table II).

**Table II:** Linear mixed-model results, fixed effects for time points, group, time points x group, and univariate effects in the neck-specific exercise with internet support (NSEIT) group and the neck-specific exercise at a physiotherapy clinic (NSE) group

Outcome	Test	F-statistic	*p*-value	ES
ACROM flexion/extension (degrees)	Time points	F(2, 112.2) = 27.250	**< 0.001**	1.39
	Group	F(1, 134.0) = 0.286	0.594	
	Time points × group	F(2, 112.3) = 3.374	**0.038**	0.48
	NSEIT time points within group	F(2, 115.06) = 22.181	**< 0.001**	1.24
	NSE time points within group	F(2, 116.587) = 8.552	**< 0.001**	0.77
ACROM lateral flexion (degrees)	Time points	F(2, 111.4) = 13.138	**< 0.001**	0.92
	Group	F(1, 133.5) = 1.311	0.254	
	Time points × group	F(2, 111.5) = 1.294	0.278	
	NSEIT time points within group	F(2, 113.697) = 6.457	**< 0.001**	0.64
	NSE time points within group	F(2, 115.16) = 8.009	**< 0.001**	0.74
ACROM rotation (degrees)	Time points	F(2, 116.5) = 29.878	**< 0.001**	1.43
	Group	F(1, 135.3) = 0.040	0.842	
	Time points × group	F(2, 116.6) = 1.351	0.263	
	NSEIT time points within group	F(2, 113.862) = 21.958	**< 0.001**	1.24
	NSE time points within group	F(2, 114.406) = 9.303	**< 0.001**	0.81
CCFT (mmHg)	Time points	F(2, 122.5) = 41.274	**< 0.001**	1.64
	Group	F(1, 133.3) = .419	0.518	
	Time points × group	F(2, 122.6) = 1.267	0.285	
	NSEIT time points within group	F(2, 115.917) = 26.353	**< 0.001**	1.35
	NSE time points within group	F(2, 118.102) = 16.329	**< 0.001**	1.05
Ventral NME (seconds)	Time points	F(2, 107.9) = 16.774	**< 0.001**	0.44
	Group	F(1, 134.6) = 0.812	0.369	
	Time points × group	F(2, 108.0) = 1.506	0.226	
	NSEIT time points within group	F(2, 353) = 7.612	**< 0.001**	0.42
	NSE time points within group	F(2, 353) = 2.101	**< 0.001**	0.22
Dorsal NME (seconds)	Time points	F(2, 101.9) = 21.490	**< 0.001**	1.30
	Group	F(1, 127.4) = 1.642	0.202	
	Time points × group	F(2, 102.0) = 0.452	0.638	
	NSEIT time points within group	F(2, 101.748) = 10.883	**< 0.001**	0.93
	NSE Time points within group	F(2, 103.579) = 11.026	**< 0.001**	0.92
Neck pain intensity before test VAS (mm)	Time points	F(2, 113.6) = 33.769	**< 0.001**	1.54
	Group	F(1, 131.7) = 0.735	0.393	
	Time points × group	F(2, 113.7) = 0.972	0.381	
	NSEIT time points within group	F(2, 107.975) = 12.413	**< 0.001**	0.96
	NSE time points within group	F(2, 108.214) = 22.347	**< 0.001**	1.29
Neck pain intensity after test VAS (mm)	Time points	F(2, 110.3) = 32.976	**< 0.001**	1.55
	Group	F(1, 132.1) = 0.077	0.782	
	Time points × group	F(2, 110.4) = 0.302	0.740	
	NSEIT time points within group	F(2, 105.22) = 19.122	**< 0.001**	1.21
	NSE time points within group	F(2, 108.947) = 14.188	**< 0.001**	1.02

NSEIT: neck-specific exercises with internet support; NSE: neck-specific exercises at a physiotherapy clinic; test: tests of fixed effects in time points (baseline, 3- and 15-month follow-ups), group (NSEIT/NSE), time points x group, and test of univariate effects (time points within group, baseline, 3- and 15-month follow-ups) in NSEIT and NSE; ES: effect size, Cohen’s d; CCFT: cranio-cervical flexion test; NME: neck muscle endurance; ACROM: active range of motion; VAS: visual analogue scale; NSEIT: neck-specific exercises with internet support; NSE: neck-specific exercises supervised at a physiotherapy clinic; VAS: visual analogue scale 0-100 mm, 0 = no pain, 100 = worst imaginable pain.

Significance of outcomes is shown in bold.

## RESULTS

**Fig. 1:**
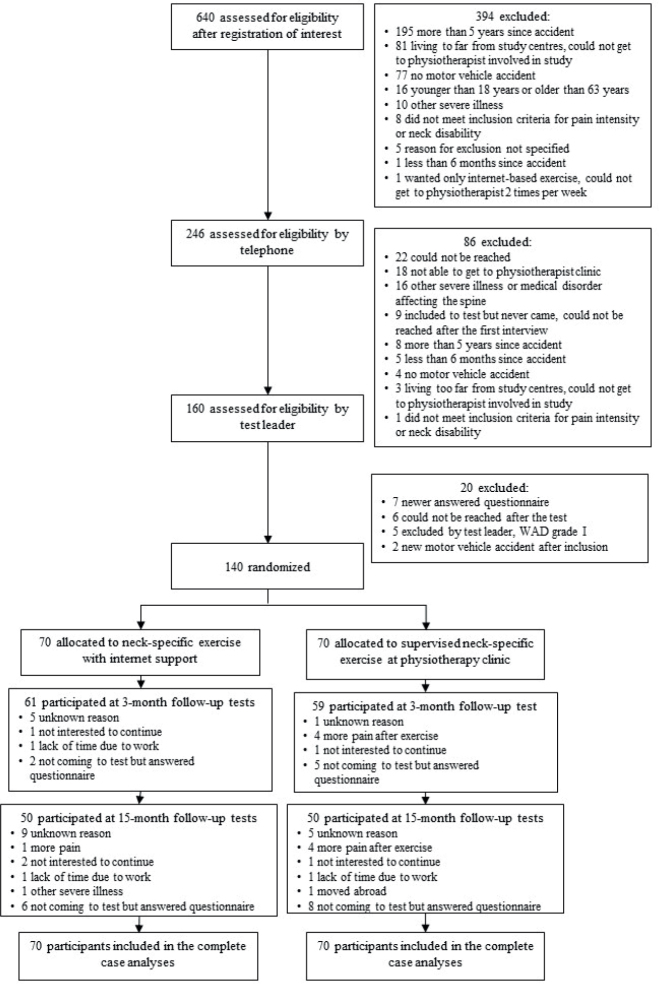
Flowchart.

In total, 140 participants were included and randomized to either the NSEIT (*n* = 70) group or the NSE (*n* = 70) group (Fig. 1). In the NSEIT group, 61 (87%) and 50 (71%) participants were followed up during 3- and 15-month tests, respectively. In the NSE group, 59 (84%) and 50 (71%) participants were followed up during 3- and 15-month tests, respectively. There were no significant differences in baseline variables between the groups (see Table I, *p* ≥ 0.15). Participants in the NSE group reported higher compliance with exercise (*n* = 59; 94%) than the NSEIT group (*n* = 46; 75%, *p* < 0.01) during the 12-week intervention period. The missing pattern analyses regarding completers/non-completers (individuals who drop out at either 3- or 15-month follow-up or both) revealed no significant differences in any outcomes between the NSEIT and NSE groups (*p* > 0.09). No adverse events were reported.

### Between-group differences

Results from the linear mixed model analyses are indicated in Table II. Baseline and 3- and 15-month follow-up data in ACROM, CCFT, NME and neck pain are listed in Table III.

**Table III:** Outcomes and between-group effects for each treatment group at baseline and at 3- and 15-month follow-ups, and within-group effects at 3- and 15-month follow-ups

Group	Time points, mean (95% CI); *p*-value			Within-group change, mean (95% CI); *p*-value	
	Baseline	3 months	15 months	3 months – baseline	15 months – baseline
ACROM flexion/extension					
NSEIT	90.4 (84.2 to 96.6)	106.2 (99.9 to 112.5)	108.1 (101.3 to 114.9)	15.8 (9.2 to 22.4); ***p* < 0.001**,	17.7 (10.0 to 25.4); ***p* < 0.001**
NSE	97.3 (91.2 to 103.5)	103.1 (96.7 to 109.5)	110.4 (103.7 to 117.2)	5.8 (−0.9 to 12.5); *p* = 0.115	13.1 (5.4 to 20.8); ***p* < 0.001**
Between-group effects	−7.0 (−15.7 to 1.8); *p* = 0.117	3.1 (−5.9 to 12.0); *p* = 0.499	−2.3 (−11.9 to 7.2); *p* = 0.629	10.0 (2.4 to 17.7); ***p* = 0.011**	4.6 (−4.3 to 13.5); *p* = 0.305
ACROM lateral flexion					
NSEIT	68.0 (64.3 to 71.8)	74.2 (70.5 to 78.0)	73.7 (69.6 to 77.9)	6.2 (1.8 to 10.6); ***p* = 0.003**	5.7 (1.1 to 10.4); ***p* = 0.010**
NSE	65.3 (61.5 to 69.0)	69.6 (65.8 to 73.4)	72.9 (68.7 to 77.1)	4.4 (−0.1 to 8.8); *p* = 0.057	7.7 (3.0 to 12.3); ***p* < 0.001**
Between-group effects	2.7 (−2.5 to 8.0); *p* = 0.306	4.6 (−0.8 to 9.9); *p* = 0.092	0.8 (−5.1 to 6.7); *p* = 0.787	1.8 (−3.3 to 7.0); *p* = 0.479	−1.9 (−7.3 to 3.4); *p* = 0.475
ACROM rotation					
NSEIT	104.3 (98.2 to 110.5)	120.3 (114.9 to 125.8)	124.6 (118.9 to 130.3)	16.0 (8.9 to 23.1); ***p* < 0.001**	20.3 (12.8 to 27.7); ***p* < 0.001**
NSE	109.3 (103.2 to 115.5)	119.5 (114.0 to 124.9)	122.6 (116.9 to 128.2)	10.1 (3.0 to 17.3); ***p* = 0.002**	13.2 (5.8 to 20.7); ***p* < 0.001**
Between-group effects	−5.0 (−13.7 to 3.7); *p* = 0.257	0.9 (−6.8 to 8.6); *p* = 0.822	2.0 (−6.0 to 10.1); *p* = 0.616	5.9 (−2.4 to 14.1); *p* = 0.162	7.0 (−1.6 to 15.6); *p* = 0.108
CCFT					
NSEIT	23.0 (22.1 to 23.8)	25.8 (25.0 to 26.6)	26.6 (25.8 to 27.4)	2.8 (1.7 to 4.0); ***p* < 0.001**	3.6 (2.4 to 4.9); ***p* < 0.001**
NSE	23.1 (22.2 to 24.0)	25.7 (24.9 to 26.5)	25.8 (25.0 to 26.5)	2.6 (1.4 to 3.8); ***p* < 0.001**	2.7 (1.4 to 3.9); ***p* < 0.001**
Between-group effects	−0.1 (−1.3 to 1.1); *p* = 0.845	0.1 (−1.0 to 1.3); *p* = 0.842	0.9 (−0.2 to 2.0); *p* = 0.120	0.2 (−1.1 to 1.6); *p* = 0.725	1.0 (−0.5 to 2.4); *p* = 0.178
Ventral NME					
NSEIT	31.3 (23.9 to 38.8)	53.0 (41.7 to 64.3)	54.4 (44.1 to 64.7)	21.7 (10.0 to 33.4); ***p* < 0.001**	23.1 (11.3 to 34.9); ***p* < 0.001**
NSE	32.7 (25.3 to 40.2)	46.1 (34.8 to 57.4)	44.2 (33.9 to 54.5)	13.3 (1.6 to 25.1); ***p* = 0.021**	11.5 (−0.3 to 23.3); *p* = 0.060
Between-group effects	−1.4 (−11.9 to 9.1); *p* = 0.791	6.9 (−9.1 to 22.9); *p* = 0.392	10.2 (−4.3 to 24.8); *p* = 0.167	8.3 (−5.2 to 21.9); *p* = 0.224	11.7 (−2.0 to 25.3); *p* = 0.093
Dorsal NME					
NSEIT	145.1 (106.4 to 183.7)	219.2 (172.1 to 266.2)	227.7 (175.2 to 280.2)	74.1 (32.9 to 115.3); ***p* < 0.001**	82.6 (28.6 to 136.5); ***p* < 0.001**
NSE	107.5 (69.4 to 145.6)	169.8 (123.3 to 216.4)	203.4 (151.0 to 255.9)	62.3 (21.9 to 102.7); ***p* < 0.001**	95.9 (42.3 to 149.5); **< 0.001**
Between-group effects	37.6 (−16.6 to 91.7); *p* = 0.173	49.3 (−16.8 to 115.5); *p* = 0.142	24.2 (−50.0 to 98.4); *p* = 0.519	11.8 (−35.3 to 58.8); *p* = 0.621	−13.4 (−75.3 to 48.6); *p* = 0.670
Neck pain before test					
NSEIT	32.9 (27.7 to 38.0)	25.1 (19.2 to 31.1)	19.4 (14.2 to 24.6)	−7.8 (−15.0 to −0.5); ***p* = 0.030**	−13.4 (−20.1 to −6.8); ***p* < 0.001**
NSE	39.0 (33.9 to 44.1)	25.8 (19.8 to 31.9)	20.8 (15.6 to 26.0)	−13.2 (−20.5 to −5.9); ***p* < 0.001**	−18.2 (−24.9 to −11.6); ***p* < 0.001**
Between-group effects	−6.2 (−13.4 to 1.1); *p* = 0.094	−0.7 (−9.2 to 7.7); *p* = 0.863	−1.3 (−8.7 to 6.0); *p* = 0.719	5.4 (−2.9 to 13.8); *p* = 0.202	4.8 (−2.8 to 12.5); *p* = 0.214
Neck pain after test					
NSEIT	50.2 (44.4 to 56.0)	34.5 (27.8 to 41.1)	30.4 (23.9 to 37.0)	−15.8 (−23.8 to −7.8); ***p* < 0.001**	−19.8 (−27.8 to −11.8); ***p* < 0.001**
NSE	49.8 (44.0 to 55.5)	35.0 (28.4 to 41.7)	33.4 (26.9 to 40.0)	−14.7 (−22.7 to −6.7); ***p* < 0.001**	−16.3 (−24.4 to −8.3); ***p* < 0.001**
Between-group effects	0.5 (−7.7 to 8.6); *p* = 0.909	−0.6 (−10.0 to 8.8); *p* = 0.905	−3.0 (−12.2 to 6.3); *p* = 0.526	−1.0 (−10.3 to 8.2); *p* = 0.823	−3.4 (−12.7 to 5.8); *p* = 0.463

ACROM: active cervical range of motion (degrees); CCFT: cranio-cervical flexion test (mmHg); NME: neck muscle endurance (seconds); neck pain intensity; Neck pain was measured before the first test (ACROM flexion/extension) and after the last test (Dorsal NME) using the visual analogue scale (0 = no neck pain, 100 = worst imaginable neck pain); NSEIT: neck-specific exercises with internet support; NSE: neck-specific exercise at a physiotherapy clinic. All values are mean (95% confidence interval). Between-group effects: differences between the NSEIT and NSE groups, and a positive value favours NSEIT.

Significance of outcomes is shown in bold.

There was no significant main effect of group (NSEIT/NSE) in ACROM (flexion/extension: F = 0.29, *p* = 0.594; lateral flexion: F = 1.3, *p* = 0.254; rotation: F = 0.04, *p =* 0.842), in the neck muscles tests (CCFT: F = 0.52, *p* = 0.419; ventral NME: F = 0.81, *p* = 0.369; dorsal NME: F = 1.64, *p* = 0.202), or in neck pain intensity (immediately before: F = 0.74, *p* = 0.393; and after the tests: F = 0.07, *p* = 0.782).

### Interaction effects

There was a significant group-by-time interaction effect in ACROM flexion/extension (F = 3.4, *p* = 0.038, ES = 0.48). The NSEIT group improved between baseline and both 3- and 15-month follow-ups (*p* < 0.001). The NSE group improved from baseline to 15-month follow-up (*p* < 0.001) but not to 3-month follow-up (*p* = 0.115). There were no significant group-by-time interaction effects in ACROM lateral flexion or rotation, CCFT, ventral and dorsal NME, or neck pain intensity (before or after the tests).

### Within-group effects

Within-groups results from the linear mixed model analyses are given in Table II. Within-group effects are indicated in Table III. There were significant within-group effects between baseline and 3- and 15-month follow-ups in the NSEIT group, with improvements in ACROM, CCFT, ventral and dorsal NME, and neck pain intensity (*p* < 0.03). There were significant improvements over time in the NSE group except from baseline to 3-month follow-up in ACROM flexion/extension (*p* = 0.115) and lateral flexion (*p* = 0.057), and between baseline and 15-month follow-up in ventral NME (*p* = 0.06).

## DISCUSSION

In individuals with persistent WAD grades II and III, we found that an internet-delivered neck-specific exercise programme with 4 physiotherapist visits improved neck muscle endurance and cervical range of motion to the same extent as supervised NSE at a physiotherapy clinic. Cervical range of motion and deep and global neck muscle endurance were significantly improved for most of the outcomes in both groups. Neck pain intensity measured immediately after the dorsal NME test was significant decreased in both groups after 3 months of neck-specific exercises, and the results were sustained at 15-month follow-up, indicating improvement in tolerating loading to the neck.

The effects of NSEIT on ACROM, CCFT, and NME were at least as beneficial as NSE at 15-month follow-up. The recommendation was daily exercises during the first weeks, with individual progression to loaded exercises 3 times a week. Despite the NSEIT group reporting lower compliance with the exercise intervention compared with the NSE group, both groups improved. This result indicates that a lower dosage of exercises than was recommended in the present study seems to have been sufficient to achieve the same improvements as in the NSE group. Internet-based interventions have shown effects in non-specific neck pain (29–30), but to our knowledge no studies have included participants with WAD. The review by Zou et al. (29) reported improvements in pain and disability in favour of telerehabilitation but the quality of the included studies was mediocre. Both synchronous and asynchronous internet-based exercises gave similar improvements in pain and disability (29) but with short follow-up periods. Patients with WAD were not included and a functional test (ACROM) was evaluated in only one study (30). Internet-based interventions seem to have an effect in patients with neck pain, but further high-quality, large-sample studies are needed that also include WAD to validate the effects found in the present study.

The facet joints and capsules are suggested to be a source of ongoing pain and disability (31) in approximately 29% of WAD patients (32). Together with altered neck muscle function, seen both in individuals with WAD and in those with non-specific neck pain (33, 34), the ability to regulate and initiate neck movement may be affected. This may cause problems maintaining a stable cervical base for the postural control of the neck, as well as sustaining load to the neck (35) in daily activities, leading to negative spirals with continued disability and pain. The neck-specific exercises in the NSEIT and NSE groups were initially targeted at facilitating deep neck muscle activity. Improved neuromuscular control of the cervical spine and increased endurance in the deep neck muscles during the first weeks of exercises were thereby supposed to lead to decreased pain from the cervical joints and capsules. During the initial exercise period, the exercise programme was designed to avoid activation of painful superficial neck muscles, as these muscles often have increased activity and delayed ability to relax after activation in individuals with neck pain (36). This in turn enhances the ability to successfully progress the exercise programme, and gradually improves global neck muscle endurance without increased neck pain intensity.

During the first week, exercises were performed with only eye shifts in the direction of neck flexion, extension, and rotation, to avoid increased pain. Thereafter, the participants continued with low-resistance isometric muscle activation in the same direction as the eye shift, with progression to performing these exercises in a seated position. Eye movements activate neck muscles but the activation is altered in chronic WAD (37). In the present study, ACROM in all 3 plans increased in both NSEIT and NSE groups despite only 1 cervical movement exercise being included: cervical rotation with a rubber band. All other neck-specific exercises during the 12-week programme were isometric without active head movements. The initial exercises with eye shift may have paved the way for improvement in ACROM in all plans, as the muscles being activated are related to neck muscles (37) involved in cervical movement. In combination with enhanced muscle endurance, the individual thereby has the possibility to move their neck and head without increased pain in daily activities.

The addition of 2 ventral neck exercises in the present study seems to have been beneficial, as ventral NME improved to a higher extent compared with our previous RCT (22). An enhanced understanding of how altered neck muscle function after whiplash injury can be improved is essential in order to develop an effective neck exercise programme. Up to the 3-month follow-up in the present study, ventral NME increased by 67% in NSEIT and 40% in NSE. Dorsal NME increased by 51% and 58% respectively, and the improvements in ventral and dorsal NME were sustained to 15-month follow-up. The results show that the neck-specific exercise programme had the intended effect, i.e., it increased neck muscle endurance, important for reduction in neck pain (14). Both the NSEIT and NSE groups also showed significantly less neck pain immediately after neck muscle endurance testing at 3- and 15-month follow-up, despite the fact that the test time with a load was increased by more than 50%. This finding is interpreted as increased loading capacity of the neck after 3 months of neck-specific exercises also seen in previous studies (16, 22).

Few studies have investigated the effects of exercises on ACROM, CCFT, or NME in chronic WAD (8, 14, 22). Advice was equally as effective as a comprehensive exercise programme in improving ACROM (8), and improvement in NME was important for neck pain reduction (14). However, only 27–48% achieved normal values in ACROM and NME after 3 months of neck-specific exercises (22), indicating the importance of improving exercise interventions for chronic WAD. There is still no consensus on the best exercise intervention for chronic WAD (7) and there is a lack of high-quality studies investigating whether one exercise type is better than another. For non-specific neck pain, motor-control exercises, Pilates, resistance training, traditional Chinese exercise, and yoga were more effective than controls, but no large differences were found between the exercise types (38). Strengthening exercises were effective for pain reduction at short term follow-up in non-specific neck pain (39), but showed increased pain intensity in chronic WAD after the exercise period (9).The included behavioural components are intended to provide an understanding of the importance of having a plan for neck pain relapse. The results in neck function were, to a great extent, sustained 1 year after the end of the intervention period. Participants in the NSEIT and NSE groups described having increased knowledge regarding WAD, a stronger neck, and fewer symptoms (40), and both the information and the exercises thus seem to have been important. It was also important to be acknowledged and to have a plan for rehabilitation (40). The enhanced understanding about WAD and pain processes may have been related to the ability to perform the exercises.

Chronic moderate (grade II) and severe (grade III) WAD is generally regarded as difficult to treat. In light of this, the results of this study showed promising results because ACROM, CCFT, NME, and neck pain all improved in individuals with chronic WAD grade II and III after 3 months of NSE or NSEIT.

### Limitations

The present study has several limitations. The power of the study was calculated based on the primary outcome Neck Disability Index (NDI). When secondary outcomes are reported, more statistical tests are conducted on the same dataset and there is a risk of false significant effect (type I error). However, linear mixed-model analyses of secondary outcomes showed no significant main effect of group, and the effect over time in the secondary outcomes was highly significant. Limitations with the cranio-cervical flexion test are that the scale has only 5 levels and it could be difficult to differentiate results. Tenderness and pain in neck structures may also affect the test. However, the test leader has not reported a problem with pain or tenderness during the CCFT in their test reports. Cervicothoracic kyphosis may affect the test if the cuff must be filled more, and it becomes harder. Layers of towel were placed under the head if necessary to achieve a neutral position of the neck. Patients with cervicothoracic kyphosis lay on 1 or several towels. Thereby the towels filled the gap between the neck and the examination table and the cuff was not filled more. Moreover, both education and exercises were included in the neck-specific exercise programme, but no consensus could be drawn regarding whether – and, if so, to what extent – education or exercises are important for improving neck function. The dropout rate to the 15-month follow-up test was high (29%) in both groups. However, the missing pattern analysis revealed no significant differences between completers/non-completers.

### Conclusion

There were no group differences between the NSEIT and NSE groups in ACROM, CCFT, NME, or neck pain before and after these tests. Both exercise groups – NSEIT with 4 physiotherapist visits and NSE supervised by a physiotherapist twice a week for 12 weeks – improved in ACROM, CCFT, NME, and pain to the same extent. Neck pain intensity measured immediately after the dorsal NME test was significantly decreased in both groups after 3 months of neck-specific exercises, and the results were sustained at 15-month follow-up, indicating improvements in tolerating loading to the neck.
